# Efficacy of an Online Meaning‐Centered Psychotherapy in Caregivers of Advanced Cancer (eMCP‐C): A Mixed‐Method Pilot Randomized Controlled Trial

**DOI:** 10.1002/pon.70420

**Published:** 2026-03-10

**Authors:** Naomi Takemura, Arthur Cheuk‐Man Li, Wing Lok Chan, Sau Fung Yu, Vivian Weiqun Lou, Daniel Yee‐Tak Fong, Jessica Kang Qi Lee, Janelle Yorke, Allison J. Applebaum

**Affiliations:** ^1^ School of Nursing The Hong Kong Polytechnic University Hong Kong SAR China; ^2^ JC STEM Lab of Digital Oncology Care Enhancement Hong Kong SAR China; ^3^ LKS Faculty of Medicine The University of Hong Kong Hong Kong SAR China; ^4^ Department of Clinical Oncology LKS Faculty of Medicine The University of Hong Kong Hong Kong SAR China; ^5^ School of Nursing LKS Faculty of Medicine The University of Hong Kong Hong Kong SAR China; ^6^ Department of Social Work and Social Administration Faculty of Social Sciences The University of Hong Kong Hong Kong SAR China; ^7^ Sau Po Centre on Ageing The University of Hong Kong Hong Kong SAR China; ^8^ Division of Nursing Midwifery & Social Work School of Health Sciences University of Manchester Manchester UK; ^9^ Brookdale Department of Geriatrics and Palliative Medicine Steven S. Elbaum Family Center for Caregiving Icahn School of Medicine at Mount Sinai New York New York USA

**Keywords:** advanced cancer, caregivers, Chinese, meaning‐centered, psychotherapy

## Abstract

**Background:**

Rising advanced cancer incidence globally places profound existential distress and psychological burden on family caregivers. We culturally adapted Meaning‐Centered Psychotherapy for Cancer Caregivers (MCP‐C) from Western to Chinese context, targeting existential distress through meaning‐making processes.

**Aims:**

This pilot randomized controlled trial aimed to examine the feasibility, acceptability, and preliminary effects of an online MCP‐C (eMCP‐C) among caregivers of patients with advanced cancer.

**Methods:**

Caregivers of patients with advanced cancer, experiencing distress related to caregiving, were randomly assigned (1:1) to eMCP‐C or enhanced usual care groups. Over 7 weeks, participants received weekly individual session of eMCP‐C focusing on meaning making and coping with caregiving, whereas enhanced usual care group received resources for mental health treatment and targeted referrals for specific distress problems. Feasibility outcomes were assessed through questionnaires and semi‐structured interviews at 7‐week. Efficacy outcomes were assessed by questionnaire at baseline, 7, and 19 weeks.

**Results:**

Forty‐six caregivers enrolled (26–79 years old), among which thirty‐seven (80%) completed the study. Intervention attendance, retention rates, and participant satisfaction were satisfactory. Depression, anxiety, meaning, peace, and self‐esteem improved from baseline to immediate and 3‐month post‐intervention in intervention group. Qualitative analysis revealed three key therapeutic mechanisms: (1) Therapeutic Alliance as a Catalyst for Intrapersonal and Interpersonal Healing, (2) Meaning‐making Through Acceptance and Action, and (3) Empowering Through Affirmation and Self‐Determined Action.

**Conclusions:**

For Chinese caregivers of patients with advanced cancer, eMCP‐C is feasible, well‐accepted, and showed preliminary benefits in psychological distress, meaning, peace, and self‐esteem, compared to an enhanced usual care group.

## 1 Background

Cancer incidence continues to increase worldwide, where more than half of the three most prevalent cancers are diagnosed at an advanced stage [1]. While therapeutic advances have extended survival for patients with advanced disease, the primary responsibility for daily care provision rests with family members and informal caregivers operating beyond the scope of formal healthcare systems [2, 3]. Family caregivers are relatives or friends who provide unpaid physical, emotional, and social support and demanding tasks [4, 5, 6]. This contributes to high rates of psychological distress, affecting up to 90% of caregivers throughout the illness trajectory [7, 8, 9]. Such distress persists beyond diagnosis, frequently manifesting as heightened death anxiety and distinct existential difficulties, including guilt, anticipatory grief, and despair regarding life after the patient's death [10, 11]. Collectively, these factors lead to existential distress in approximately one‐third of this population, characterized by loss of dignity, despair, death anxiety, lack of self‐efficacy, and lack of meaning [12]. Existential distress represents a core driver of caregiver burden and is strongly associated with adverse outcomes such as depression and feelings of powerlessness [13]. Furthermore, social pressures to maintain hope and positivity further inhibit open expression of distress and impede help‐seeking behaviors [14]. Consequently, caregivers of people with advanced cancer constitute a highly vulnerable group in need of tailored support.

Existing interventions for this population primarily focus on psychoeducational or problem‐solving approaches, which often lack a dedicated focus on addressing these underlying existential concerns [15, 16]. Meaning‐centered interventions address this gap by explicitly targeting existential distress through meaning‐making processes grounded in existential philosophy and Frankl's logotherapy theory. These approaches facilitate the discovery of purpose and resilience amid suffering, fostering psychological growth and well‐being [17]. Among these interventions, Meaning‐Centered Psychotherapy for Cancer Caregivers (MCP‐C) has been specifically adapted to help caregivers connect with four sources of meaning: historical, attitudinal, creative, and experiential sources of meaning [18]. Western studies with glioblastoma multiforme caregivers demonstrate MCP‐C's feasibility and potential benefits, including enhanced personal meaning, benefit finding, spiritual well‐being, and reduced depressive symptoms [13, 19]. However, meaning‐making processes are culturally contextualized. Cultural adaptation maximizes intervention relevance by aligning content with the target population's values, beliefs, and context [20]. Evidence reveals that culturally tailored interventions enhance receptivity to health information and programmes, ultimately improving effectiveness and outcomes [21, 22]. Hence, our team has culturally and linguistically adapted MCP‐C in Chinese context through rigorous translation and conceptual equivalence testing [23].

The primary aim of the study was to explore the feasibility and acceptability of conducting a powered RCT comparing culturally adapted MCP‐C with control group among Chinese caregivers of patients with advanced cancer. We hypothesized that culturally adapted MCP‐C would be feasible and acceptable in this population, and would demonstrate preliminary efficacy in reducing psychological distress and caregiver burden, while enhancing meaning in life and spiritual well‐being.

## 2 Method

### 2.1 Study Design

This study was an assessor‐blinded, two‐arm RCT employing a sequential exploratory mixed‐methods approach to evaluate feasibility and preliminary effect on existential distress among caregivers of patients with advanced cancer. Participants were randomly allocated to intervention and control group at a 1:1 ratio. The randomized allocation sequence was generated using Random Allocation Software 2.0 by an independent research personnel. We used block randomization of randomized size 4–8. Feasibility outcomes were evaluated through mixed‐methods at the 7‐week follow‐up (i.e., post‐intervention). Data on effect outcomes were collected and assessed at baseline, as well as the 7‐ (T1) and 19‐week follow‐ups (T2). Individual semi‐structured interviews were conducted at 7‐week follow‐up. This study adhered to the Consolidate Standard of Reporting Trials (CONSORT) statement for reporting.

### 2.2 Participants and Setting

Participants were eligible if they were (i) aged 18 or above; (ii) current family caregiver to a patient diagnosed of stage III or IV cancer; (iii) able to read or understand Chinese; (iv) presented with caregiving distress as evidence by a score of ≥ 4 on the Distress Thermometer (DT) with distress related to caregiving. Exclusion criteria included pre‐existing psychotic condition or cognitive affliction which might, respectively, confound the outcome evaluation or hamper the participation. Potential participants were recruited from the Department of Clinical Oncology at a government‐funded hospital in Hong Kong. Written informed consent was obtained from eligible participants before study commencement.

### 2.3 Sample Size

The sample size was calculated to ensure reliable estimation of the feasibility outcomes, including recruitment rate, retention rate, intervention attendance, and satisfaction. For rates, we anticipated them as 70%. Taking 15% margin of error in a 95% confidence interval, we need 23 caregivers per group. For estimating the satisfaction level, we took a moderate standardized margin of error of 0.3, which also required 23 caregivers in the intervention group. Therefore, we recruited a total of 46 caregivers in this study.

### 2.4 Interventions

#### 2.4.1 Online Meaning‐Centered Psychotherapy for Cancer Caregivers (eMCP‐C)

The eMCP‐C is a 7‐session individual intervention, focusing on themes related to meaning‐making and caregiving (Supporting Information S1: Appendix 1). Every session uses didactics, experiential exercises, and psychotherapeutic techniques to facilitate the caregivers to find meaning in the challenging caregiving process and to mobilize it as the internal resource to sustain the care motivation and resilience. Each session addresses specific aspects of meaning‐making in caregiving, with the aim of supporting caregivers to reflect on their encounter along their journey. The themes of each session are described as follows, Session 1‐Concepts and Sources of Meaning provides an overview of meaning and caregiving, allowing participants to share their caregiving journey. Session 2‐ Identity Before and After Becoming a Caregiver explores on the impact of caregiving on the participants' identities and how their roles have evolved. Session 3‐ Historical Sources of Meaning delves into the critical elements of caregivers' past, present, and future legacies, many of which may be connected to the caregiving role. Examples of narrative content include past experiences of providing care or observing others providing care, past experiences of illness or loss, and family values associated with an ethic of care, taking pride in caregiving, and setting examples for future generation. Session 4‐ Attitudinal Sources of Meaning examines the ways caregivers choose to face limitations and challenges. Examples of narrative content include caregivers' decision to provide care and engage fully in the relationship with the care recipient despite the possibility of its ending, as well as how caregiver faces the resulting limitations. Session 5‐ Creative Sources of Meaning focuses on how caregivers create meaning and take responsibility for their own lives, including engaging in the caregiving role and attending to their own needs. Examples of narrative content include courageous engagement in caregiving, self‐care, and discussions of the existential and neurotic guilt as indicators of deficient self‐care. Session 6‐ Experiential Sources of Meaning centers on the meaning derived through the five senses and through love, beauty and humor. Session 7‐ Transitions provides an opportunity for participants to review the sources of meaning and reflect on how they can use these as resources moving forward. Caregivers are assigned readings and homework exercises specific to each session's theme and which guide discussions in subsequent sessions. All eMCP‐C sessions were delivered via secured virtual platform (Zoom) to ensure flexibility in scheduling.

#### 2.4.2 Fidelity of the Intervention

A research assistant with psychology background was employed as the interventionist of this study. She has received professional training in the rationale, principles, and implementation of eMCP‐C from the clinical psychologist in the team. All sessions will be audio‐taped, with prior consent obtained from participants. A random sample of 30% of the sessions were evaluated and rated for treatment integrity. Adherence ratings were an established treatment integrity coding form utilized by previous studies. There were five items of content rated on a Yes/No scale and five items of process rated on a 0–2 scale, adding to a total score of 15.

#### 2.4.3 Enhanced Usual Care

Participants in the enhanced usual care group received resources for mental health treatment and targeted referrals specific to problems identified on the DT such as practical problems, family problems, and physical problems [19]. This approach has been adopted in previous studies in MCP in cancer patients and caregivers [24]. Participants who scored ≥ 8 on DT were offered to be referred to our team's clinical psychologist.

### 2.5 Measures

Sociodemographic characteristics were collected via a self‐administered questionnaire on sociodemographic, household information, and caregiving‐related information.

### 2.6 Primary Outcomes

Feasibility and acceptability outcomes were assessed by both quantitative and qualitative outcomes. *Quantitative outcomes* included eligibility rate, recruitment rate, retention rate, intervention adherence, and satisfaction with treatment measured using a questionnaire rating on four key domains, (1) usefulness of treatment, (2) opinion of the therapist, (3) perceived improvement, and (4) likelihood to recommend the treatment to others, on a 10‐point scale, with higher scores indicating higher satisfaction. Details of the benchmarks were described in Supporting Information S1: Appendix 2. *Qualitative outcomes* were evaluated via semi‐structured interviews, aiming (i) to explore participants' perceptions of and satisfaction with the intervention, (ii) to understand their perceived effects of the intervention (the way they adapt to the process of caregiving, the way they feel about caregiving, and their perceived well‐being), (iii) to identify elements of the intervention viewed as helpful/unhelpful for further intervention modifications, and (iv) to explore the conditions affect their engagement in e‐MCP. The interviews were audio‐recorded, transcribed, and anonymized. To include a diverse array of participants in the intervention, maximum variation sampling was used to incorporate participants characteristics in terms of sociodemographic factors (e.g., sex), kinship with the care recipient (spouse/siblings) and adherence rates (high/low).

### 2.7 Secondary Outcomes

These outcomes were evaluated at baseline (T0), T1 and T2 in both arms.

Distress Thermometer is a single‐item visual analog scale with a 0–10 range [25]. The National Cancer Center Network Clinical Practice Guidelines for Distress Management cutoff of ≥ 4 was used for identifying clinically significant psychological distress [26].

Psychological well‐being was measured by the Hospital Anxiety and Depression Scale (HADS), which consists of two subscales: anxiety and depression. Each subscale has 7 items rated on a 4‐point scale. A cut‐off score of ≥ 8 on either subscale indicates anxiety or depression [27]. HADS has been validated in a Chinese cancer population [28].

Meaning in life was measured by the Meaning in Life Questionnaire (MLQ), which consists of 10 items assessing the level of experienced meaning in life (presence) and desire to search for meaning in life (search). Each item is rated on a seven‐point Likert scale from 1 (absolutely untrue) and 7 (absolutely true). The Chinese version of MLQ has been validated among caregivers in Hong Kong [29].

Spiritual well‐being was measured by the Functional Assessment of Chronic Illness Therapy‐Spiritual Well‐Being Scale (FACIT‐Sp‐12) [30]. It consists of subscales that measure faith, meaning of life, and sense of peace. Each item rated on a 5‐point Likert scale ranging from 0 (nothing) to 4 (very much), with higher scores denoting higher spiritual well‐being levels. The Chinese version of FACIT‐Sp‐12 is available on their official website [31].

*Caregiver burden* was measured by Caregiver Reaction Assessment (CRA), which consists of 24 items assessing caregiving burden of the family caregivers. Each item is rated on a five‐item Likert scale, with higher score indicating a higher level of caregiving burden, except for the self‐esteem subscale where a higher score indicating a lower level of burden. CRA has been validated in a caregivers of Chinese cancer population [32].

*Benefit finding* was measured by the Benefit Finding Scale, which consists of 17 items evaluating insights of the positive contributions resulting from the process or experience of coping with cancer. Each item is rated on a 5‐point Likert scale, with higher scores indicating greater benefit finding. The Chinese version has been validated in Chinese family caregivers of cancer patients [33].

### 2.8 Statistical Analysis

All statistical analyses were conducted using SPSS 28.0. Feasibility outcomes were summarized by 95% confidence intervals. Intention‐to‐treat analysis was performed using a linear mixed‐effects model, which includes subjects with missing values at follow‐up if they had observed baseline measurements, to compare the efficacy outcomes between the two groups (MCP‐C and control) at different time points. The persistence of the intervention effect over time will be examined by incorporating a group × time interaction. All semi‐structured interviews were audio‐recorded and transcribed verbatim. Field notes will be reviewed with the transcripts during the process. Interview transcripts will be analyzed according to the thematic analysis strategy introduced by Braun and Clarke [34], using NVivo 11.0. Field notes were reviewed with the transcripts during the process. A mixed‐methods triangulation design was used to interrelate and interpret the quantitative and qualitative data to validate the results.

### 2.9 Ethics

The study was approved by the Institutional Review Board of the University of Hong Kong/Hospital Authority Hong Kong West Cluster (UW 24–031) in accordance with the Declaration of Helsinki. The trial was registered before the first person was enrolled (Chinese Clinical Trial Registry: ChiCTR2400082037). The study was conducted in accordance with Declaration of Helsinki.

## 3 Results

### 3.1 Characteristics of Participants

Table 1 presents the baseline demographic characteristics of the participants. Baseline demographic and clinical characteristics were comparable between intervention and control groups. Participants had a mean (SD) age of 52 (11) years in the intervention group and 56 (15.1) years in the control group. The majority (73.9%) were female. Most participants were married or partnered (78.3%) and had attained at least a high school education (84.8%). Half (50.0%) were employed full‐time, and slightly over half (52.2%) reported receiving no caregiving assistance from other family members. Regarding caregiving context, the majority of caregivers were caring for a spouse (65.2%), and 80.4% cohabitated with the patient. The mean (SD) daily caregiving hours were 10.7 (9.2) for the intervention group and 11.2 (9.3) for the control group.

**TABLE 1:** Baseline characteristics of participants (*n* = 46).

Characteristic	*N*	MCP‐C, *N* = 23	EUC, *N* = 23
Gender, *n* (%)	46		
Male	21	12 (52.2%)	9 (39.1%)
Female	24	11 (47.8%)	23 (60.9%)
Age	46		
Mean (SD)		52 (11)	56 (15.1)
Relationship status, *n* (%)	46		
Single	10	5 (21.7%)	5 (21.7%)
Married/partnered	36	18 (78.3%)	18 (78.3%)
Education, *n* (%)	46		
Junior high school or below	7	3 (13%)	4 (17.4%)
High school	16	8 (34.8%)	8 (34.8%)
College or above	23	12 (52.2%)	11 (47.8%)
Religious belief, *n* (%)	46		
No	31	14 (60.9%)	17 (73.9%)
Yes	15	9 (39.1%)	6 (26.1%)
Monthly household income, *n* (%)	46		
< $10,000HKD	9	4 (17.4%)	5 (21.7%)
$10,000–$29,999 HKD	9	4 (17.4%)	5 (21.7%)
≥ $30,000HKD	28	15 (65.2%)	13 (56.5%)
Employment status, *n* (%)	46		
Unemployed	3	2 (8.7%)	1 (4.3%)
Not employed ‐ retired	10	3 (13%)	7 (30.4%)
Homemaker	3	2 (8.7%)	1 (4.3%)
Paid part‐time employment	7	5 (21.7%)	2 (8.7%)
Paid full‐time employment	23	11 (47.8%)	12 (52.2%)
Household size, *n* (%)	46		
2 People family	15	6 (26.1%)	9 (39.1%)
3 People family	12	3 (13%)	9 (39.1%)
4 People family	16	12 (52.2%)	4 (17.3%)
5 People or above family	3	2 (8.7%)	1 (4.3%)
Have Children, *n* (%)	46		
No	31	10 (43.5%)	8 (34.8%)
Yes	15	13 (56.5%)	15 (65.2%)
Number of children	46		
Mean (SD)		1 (1)	1 (1)
Age of children	46		
Mean (SD)		25.2 (18.7)	33.4 (10.7)
Assistance received from other family member, *n* (%)	46		
No	24	11 (47.8%)	13 (56.5%)
Yes	22	12 (52.1%)	10 (43.5%)
Family suffers from a serious illness that needs caregiving, *n* (%)			
No	40	21 (91.3%)	19 (82.6%)
Yes	6	2 (8.7%)	4 (17.4%)
Number of care recipients	46		
Mean (SD)		1.1 (0.3)	1.1 (0.3)
Months spent providing care	46		
Mean (SD)		28.4 (24.9)	33.3 (42.1)
Hours/day spent providing care	46		
Mean (SD)		10.7 (9.2)	11.2 (9.3)
Relationship to the patient, *n* (%)	46		
Wife	17	9 (39.1%)	8 (34.8%)
Husband	13	4 (17.4%)	9 (39.1%)
Daughter/daughter‐in‐law	7	5 (21.7%)	3 (13.0%)
Son/son‐in‐law	7	5 (21.7%)	2 (8.7%)
Mother	1	0 (0%)	1 (4.3%)
Caregiver/patient cohabitation, *n* (%)	46		
With patient	37	16 (69.6%)	21 (91.3%)
With other relatives	7	5 (21.7%)	2 (8.7%)
Live alone	2	2 (8.7%)	0 (0%)

### 3.2 Primary Outcomes: Feasibility‐And Acceptability‐Related Outcomes

#### 3.2.1 Recruitment

**FIGURE 1:**
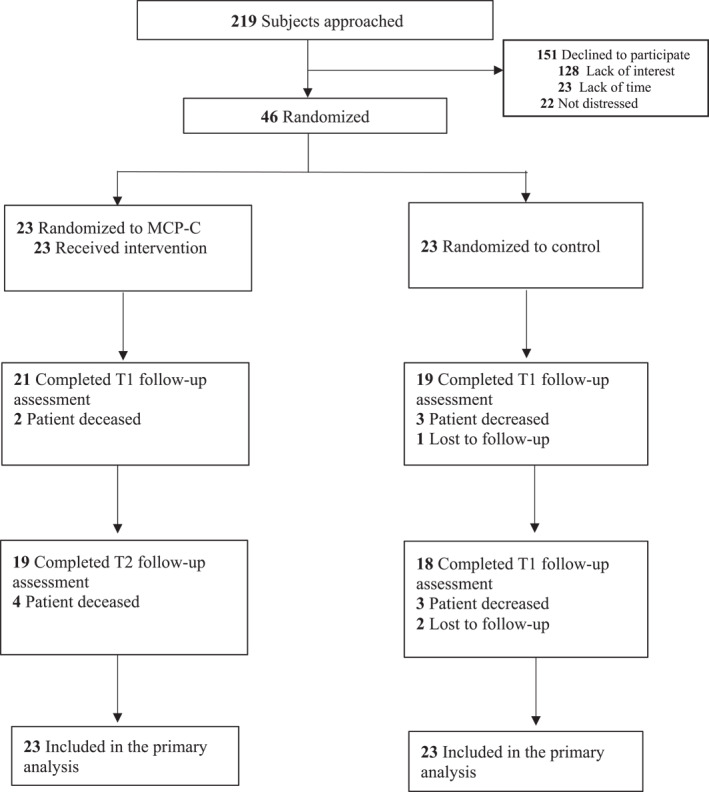
CONSORT 2020 flow diagram.

Figure 1 illustrates the recruitment flow diagram. Between July and October 2024, 219 caregivers of patients with cancer were approached and assessed for eligibility. Of these, 151 declined to participate were excluded, primarily due to lack of interest (*n* = 128), lack of time (*n* = 23), while the remaining 22 did not meet distress criteria. Forty‐six caregivers were recruited and randomized: 23 were assigned to the eMCP‐C group and 23 to the control group. The recruitment rate was 21.0% (46/219).

#### 3.2.2 Retention Rate, Attendance, Intervention Acceptability and Fidelity

Retention was 87.0% (40/46) at T1 and 80.4% (37/46) at T2. At the post‐intervention assessment (T1), six participants had withdrawn (eMCP‐C: *n* = 2; control: *n* = 4), primarily due to patient death (*n* = 5). By the 3‐month post‐intervention follow‐up (T2), cumulative withdrawals totaled nine participants (eMCP‐C: *n* = 4; control: *n* = 5). Treatment attendance was high, with 91% of caregivers completing all seven sessions. All participants reported high satisfaction (ratings ≥ 8/10) across domains including treatment usefulness, therapist opinion, perceived improvement, and likelihood of recommending the treatment. No adverse events occurred. Ratings of treatment fidelity showed that eMCP‐C adhered closely for content and process, with mean rating of 13.71.

### 3.3 Secondary Outcomes: Efficacy Outcomes

Table 2 summarizes the mixed‐effect model of all effect outcomes at T1 and T2 between intervention and control groups.

**TABLE 2:** Mixed‐effects analysis for the intervention effects (*n* = 46).

	Mean (SE)		Experimental		Control		Between‐group difference	
	Experimental (*n* = 23)	Control (*n* = 23)	Within‐group change from baseline (95% CI)	*p* value	Within‐group change from baseline (95% CI)	*p* value	Estimate (95% CI)	*p* value
Depression								
Baseline	4.87 (0.84)	6.83 (0.84)	—	—	—	—	—	—
Week 7	4.88 (1.01)	8.66 (0.89)	0.01 (−2.25, 2.28)	1.00	1.84 (−0.09, 3.76)	0.07	−3.78 (−6.46, −1.10)	0.01*
Week 19	5.27 (1.12)	8.41 (0.90)	0.40 (−2.40, 3.20)	1.00	1.58 (−0.71, 3.88)	0.27	−3.14 (−6.00, −0.29)	0.03*
Anxiety								
Baseline	8.35 (0.96)	8.52 (0.96)	—	—	—	—	—	—
Week 7	7.08 (1.14)	10.49 (1.01)	−1.27 (−3.70, 1.16)	0.61	1.97 (−0.09, 4.03)	0.07	−3.41 (−6.44, −0.39)	0.03*
Week 19	5.82 (1.26)	11.28 (1.03)	−2.53 (−5.61, 0.55)	0.14	2.75 (0.23, 5.27)	0.03	−5.46 (−8.68, −2.23)	0.001*
FACIT Sp								
Baseline	29.52 (2.18)	28.70 (2.18)	—	—	—	—	—	—
Week 7	30.99 (2.46)	27.77 (2.26)	1.47 (−2.91, 5.84)	1.00	−0.93 (−4.60, 2.75)	1.00	3.22 (−3.45, 9.89)	0.34
Week 19	32.67 (2.70)	27.90 (2.31)	3.15 (−2.82, 9.12)	0.58	−0.79 (−5.64, 4.05)	1.00	4.77 (−2.31, 11.85)	0.18
FACIT Sp‐meaning								
Baseline	11.78 (0.72)	11.04 (0.72)	—	—	—	—	—	—
Week 7	13.03 (0.83)	10.38 (0.75)	1.25 (−0.32, 2.82)	0.17	−0.66 (−1.98, 0.66)	0.66	2.65 (0.42, 4.88)	0.02*
Week 19	13.02 (0.89)	10.51 (0.76)	1.24 (−0.73, 3.20)	0.37	−0.54 (−2.12, 1.05)	1.00	2.51 (0.17, 4.85)	0.04*
FACIT Sp‐peace								
Baseline	9.52 (0.78)	8.65 (0.78)	—	—	—	—	—	—
Week 7	9.14 (0.91)	8.36 (0.82)	−0.38 (−1.46, 2.22)	1.00	−0.29 (−1.85, 1.26)	1.00	0.78 (−1.66, 3.22)	0.53
Week 19	10.99 (1.00)	8.32 (0.83)	1.47 (−0.88, 3.83)	0.38	−0.33 (−2.25, 1.58)	1.00	2.68 (0.09, 5.26)	0.04*
FACIT Sp‐faith								
Baseline	8.22 (0.97)	9.00 (0.97)	—	—	—	—	—	—
Week 7	8.82 (1.11)	9.03 (1.01)	0.60 (−1.53, 2.73)	1.00	0.03 (−1.77, 1.82)	1.00	−0.21 (−3.20, 2.79)	0.89
Week 19	8.73 (1.24)	9.10 (1.04)	0.51 (−2.42, 2.23)	1.00	0.10 (−2.25, 2.44)	1.00	−0.37 (−3.59, 2.85)	0.82
Meaning in life‐Presence								
Baseline	23.74 (1.23)	25.39 (1.23)	—	—	—	—	—	—
Week 7	25.49 (1.51)	25.28 (1.31)	1.75 (−1.72, 5.22)	0.65	−0.12 (−3.07, 2.84)	1.00	0.21 (−3.76, 4.19)	0.92
Week 19	24.47 (1.52)	24.62 (1.29)	2.73 (−0.49, 5.95)	0.12	−0.77 (−3.32, 1.78)	1.00	1.85 (−2.12, 5.82)	0.36
Meaning in life‐Search								
Baseline	20.87 (2.12)	18.13 (2.12)	—	—	—	—	—	—
Week 7	20.69 (2.81)	26.63 (2.33)	−0.18 (−8.39, 8.03)	1.00	8.50 (1.23, 15.77)	0.02	−5.94 (−13.19, 1.31)	0.11
Week 19	19.69 (3.07)	24.19 (2.34)	−1.18 (−10.21, 7.85)	1.00	6.06 (−1.57, 13.69)	0.17	−4.50 (−12.15, 3.15)	0.25
Benefit finding scale								
Baseline	45.74 (2.76)	42.52 (2.76)	—	—	—	—	—	—
Week 7	46.22 (3.40)	44.61 (2.95)	0.49 (−7.50, 8.47)	1.00	2.09 (−4.74, 8.91)	1.00	1.62 (−7.33, 10.56)	0.72
Week 19	45.80 (3.66)	44.49 (2.96)	0.06 (−9.03, 9.14)	1.00	1.97 (−5.43, 9.36)	1.00	1.31 (−8.05, 10.67)	0.78
CRA‐ impact on finance								
Baseline	10.30 (0.62)	9.74 (0.62)	—	—	—	—	—	—
Week 7	10.35 (0.72)	8.91 (0.65)	0.05 (−1.41, 1.50)	1.00	−0.83 (−2.06, 0.40)	0.30	1.44 (−0.50, 3.38)	0.14
Week 19	9.99 (0.81)	9.38 (0.67)	−0.32 (−2.29, 1.66)	1.00	−0.36 (−2.01, 1.28)	1.00	0.61 (−1.48, 2.71)	0.56
CRA‐lack of family support								
Baseline	11.65 (0.89)	11.91 (0.89)	—	—	—	—	—	—
Week 7	11.67 (1.01)	12.96 (0.92)	0.02 (−1.76, 1.81)	1.00	1.04 (−0.42, 2.50)	0.25	−1.28 (−4.01, 1.45)	0.35
Week 19	10.33 (1.10)	12.54 (0.94)	−1.32 (−3.72, 1.08)	0.53	0.63 (−1.31, 2.58)	1.00	−2.21 (−5.09, 0.67)	0.13
CRA‐ impact on health								
Baseline	11.22 (0.43)	11.78 (0.43)	—	—	—	—	—	—
Week 7	11.56 (0.51)	11.62 (0.45)	0.34 (−0.74, 1.43)	1.00	−0.16 (−1.08, 0.76)	1.00	−0.06 (−1.43, 1.30)	0.93
Week 19	11.15 (0.53)	12.35 (0.45)	−0.07 (−1.18, 1.03)	1.00	0.56 (−0.32, 1.44)	0.35	−1.20 (−2.58, 0.18)	0.09
CRA‐self‐esteem								
Baseline	28.44 (0.81)	27.87 (0.81)	—	—	—	—	—	—
Week 7	28.72 (0.93)	26.21 (0.84)	0.28 (−1.45, 2.01)	1.00	−1.67 (−3.08, −0.26)	0.02*	2.51 (0.01, 5.01)	0.049*
Week 19	28.49 (0.96)	25.91 (0.85)	0.05 (−1.97, 2.07)	1.00	−1.97 (−3.64, −0.29)	0.02*	2.58 (0.03, 5.12)	0.047*

Abbreviation: CRA, caregiver reaction assessment.

^*^ *p* < 0.05.

#### 3.3.1 Psychological Outcomes

Compared to the control group, the eMCP‐C group demonstrated potentially significant improvements in depression (mean difference [d] = −3.78; 95% CI: −6.46, −1.10) and anxiety (*d* = −3.41; 95% CI: −6.44, −0.39) at T1. These improvements were sustained at T2 for both depression (*d* = −3.14; 95% CI: −6.00, −0.29) and anxiety (*d* = −5.46; 95% CI: −8.68, −2.23).

#### 3.3.2 Spiritual Well‐Being

Potentially significant improvements were observed in the meaning subscale for the eMCP‐C group versus controls at T1 (*d* = 2.65; 95% CI: 0.42, 4.88), with effects maintained at T2 (*d* = 2.51; 95% CI: 0.17, 4.85). The peace subscale showed potentially significant improvement at T2 (*d* = 2.68; 95% CI: 0.09, 5.26). No potential significant changes occurred in the faith subscale or Meaning in Life Questionnaire scores.

#### 3.3.3 Caregiver Outcomes

Self‐esteem potentially significantly improved in the eMCP‐C group relative to controls at both T1 (*d* = 2.51; 95% CI: 0.01, 5.01) and T2 (*d* = 2.58; 95% CI: 0.03, 5.12). No potential significant improvements were observed in other Caregiver Reaction Assessment domains or on the Benefit Finding Scale.

### 3.4 Semi‐Structured Interviews

Qualitative analysis revealed three overarching therapeutic mechanisms, further elaborated through six subthemes (Table 3).

**TABLE 3:** Overview of therapeutic mechanisms and subthemes from qualitative analysis.

Overarching therapeutic mechanisms	Subthemes	Descriptions	Quotes
Therapeutic alliance as a catalyst for intrapersonal and interpersonal healing	Safe space for emotional processing and burden alleviation	Participants highlighted the intervention as a non‐hierarchical sanctuary that mitigated isolation and cognitive overload. Facilitators cultivated trust by listening reflectively and guiding self‐directed problem‐solving, contrasting sharply with advice‐driven interactions in personal relationships.	“We spoke as equals rather than in any hierarchy…, unlike others jumping to advice—what to do and how to do it.” (Participant 34, 59 years old male)
			“I didn't feel pressured with the therapist because we're not close; I worried less about affecting her (the therapist's) emotions… That let me speak freely.” (Participant 46, 34 years old male)
		Affective ventilation directly reduced perceived stress, with participants reporting a cumulative sense of relief.	“Over these 7 weeks, I felt it (eMCP‐C program) eased the burden of caregiving… I Could share [struggles] or just talk to de‐stress.” (Participant 03, 50 years old female)
	Dyadic spillover—enhanced communication and mutual understanding	Participants expressed that they gained emotional regulation skills and psychological resilience through the alliance extended into caregiver‐patient relationships. Processed distress reduced interpersonal tension, fostering patient‐centered communication. This dyadic regulation effect—where the therapist's validation equipped caregivers to replicate empathy with patients—correlated with sustained distress reduction and relational harmony.	“Once you've said it out loud… your mind feels lighter… You feel happier and at ease…You get along better with the person you're caring for when you talk more.” (Participant 17, 67 years old female)
			“I got used to expressing my feelings. He (The patient) understood me more and would ask me out for a walk. Our relationship improved as misunderstandings and conflicts reduced.” (Participant 17, 67 years old female)
Meaning‐making through acceptance and action	Reconstructing meaning through attainable and sustainable acts	Participants shifted from abstract, overwhelming ideals of purpose to concrete, “micro‐meanings” embedded in daily caregiving. This recalibration empowered action despite patient volatility. By lowering the threshold for meaningful action, caregivers generated momentum through small and frequent successful experiences. As meaning became more attainable and focused on the present moment, participants experienced less overwhelming emotions.	“I learned that ‘meaning’ doesn't have to be grand; instead, it (meaning) can be personal, even somewhat individualistic, and it still counts as meaning. For example, in my heart I might hope to keep bringing happiness to my dad. That's kind of abstract, like an ultimate goal. But when I see his condition fluctuating and he gets discouraged, I feel like I can't achieve that, and I get discouraged too. Yet, meaning can be smaller, like being able to have a meal with my dad, sit together and chat—that's meaningful too. Those things are easier to do, and they keep me feeling that I'm contributing something to my family, which is meaningful.” (Participant 08, 34 years old male)
			“Before the program, I thought life's meaning had to be something big and accomplished. After 7 weeks, I realized it's not far away; you can do meaningful things every day in small ways. That's my biggest takeaway. It also changed how I look at and do things; even small acts can feel meaningful and be healing.” (Participant 09, 32 years old female)
	Peace through present‐moment acceptance	Participants cultivated deepening inner peace as the intervention progressed, anchored in acceptance, cognitive reappraisal, and intentional focus on adaptable present‐moment experiences. This shift was often facilitated by spiritual or existential frameworks that reframed suffering as part of a natural continuum.	“Some parts reminded me—like Buddhism's reflections on life's sufferings—that birth, aging, illness, and death are natural. I Realized how much I fixated on ‘psycho‐energy’ on negatives, forgetting the good. A token has two sides—you can look either way. Through exchange, the picture becomes clearer. Even in harsh reality, we adapt here and now. (Participant 34, 59 years old male)
		Another participant consciously redirected attention from perceived threats toward workable aspects of the present, enabling balanced appraisal.	“I've thought more about how to accept, let go, and free myself. She (The therapist) taught me to collect photos or write memories—these helped. It takes courage to step out after being trapped so long.” (Participant 17, 67 years old female)
Empowering through affirmation and self‐determined action	Recognizing personal strengths builds sense of worth	Participants felt that the therapist actively spotlighted their adaptive efforts, transforming overlooked daily actions into validated competencies.	“When you're doing things every day, you don't see what you're doing. Through the intervention, you realize you've done a lot. Even terms like ‘creativity’ made me think, ‘oh, that applies to me?’ it felt like a spotlight shining down—affirming I did things right.” (Participant 03, 50 years old female)
		Some expressed explicit acknowledgment of positive coping strategies reinforced identity coherence.	“The most impressive was the part about attitude. The therapist appreciated that I chose an optimistic way to face my husband's illness—not passive or sorrowful. I've practiced this (optimistic attitude) since diagnosis, so that recognition was the most impressive.” (Participant 09, 32 years old female)
	Encouraging self‐directed action through affirmation	Participants felt the non‐directive approach actively cultivated their autonomy by guiding them to develop personalized solutions aligned with their unique contexts and values. This methodology empowered participants to design meaningful relational practices that transcended conventional caregiving scripts, transforming them from passive recipients into active architects of their care approach.	“It (The eMCP‐C) helped us find our own path—you shed light on blind spots and inspired new directions.” (Participant 03, 50 years old female)
		The synergistic integration of affirmation and self‐determination amplified their sense of agency, enabling them to reframe challenges as opportunities for purposeful action. Therapists' validation of self‐initiated practices—from daily rituals to commemorative acts—reinforced their identity as competent agents capable of creating meaning within adversity.	“By discussing future outlook and how to pass things on, it (the eMCP‐C) pushed further—asking what concrete things I could do to extend meaning… There can be sweetness within hardship.” (Participant 46, 34 years old male)

#### 3.4.1 Theme 1: Therapeutic Alliance as a Catalyst for Intrapersonal and Interpersonal Healing

Participants expressed that the facilitative relationship—characterized by equality, empathy, and confidentiality—served as a foundational catalyst, enabling both intrapersonal emotional processing and interpersonal relational improvements. This alliance not only provides immediate psychological relief but also sustained reductions in caregiver distress, while fostering healthier dynamics with care recipients.

#### 3.4.2 Theme 2: Meaning‐Making Through Acceptance and Action

Participants found the intervention facilitated a transformative process where caregivers reconstructed purpose by embracing attainable actions and cultivated inner peace through existential reappraisal and present‐moment acceptance. This dual pathway enhanced spiritual well‐being and reduced distress.

#### 3.4.3 Theme 3: Empowering Through Affirmation and Self‐Determined Action

Participants felt eMCP‐C catalyzed their empowerment by strategically combining strength‐based validation with non‐directive facilitation. This dual approach fostered self‐worth through recognized competence while restoring agency via self‐generated solutions, collectively reducing hopelessness and reinforcing mastery.

## 4 Discussion

This study was the first study to evaluate the feasibility, acceptability, and preliminary effect of eMCP‐C, the first culturally adapted intervention specifically designed to reduce existential distress among Chinese cancer caregivers. The eMCP‐C demonstrated satisfactory feasibility, evidenced by high treatment adherence and participant satisfaction rates. Furthermore, eMCP‐C showed promising preliminary efficacy, yielding statistically significant reductions in symptoms of depression and anxiety, alongside significant improvements in meaning and self‐esteem among Chinese caregivers of patients with advanced cancer. Qualiative analysis revealed three key therapeutic mechanisms: (1) Therapeutic Alliance as a Catalyst for Intrapersonal and Interpersonal Healing, (2) Meaning‐making Through Acceptance and Action, and (3) Empowering Through Affirmation and Self‐Determined Action.

All participants expressed satisfaction with the seven‐session eMCP‐C program, particularly valuing the facilitative therapeutic relationships and the intervention's role as a non‐hierarchical sanctuary. The online delivery format further enhanced acceptability by accommodating caregivers' logistical and caregiving schedule constraints. High completion and retention rates suggest participants perceived cumulative benefits—including growing senses of meaning, peace, and empowerment—that motivated continued engagement.

Though preliminary in nature, it was encouraging that seven sessions of eMCP‐C significantly alleviated symptoms of both depression and anxiety among caregivers of patients with advanced cancer, with benefits sustained from immediate post‐intervention through the 3‐month follow‐up. This outcome is particularly significant given the high prevalence of psychological distress in this population within advanced disease settings, where caregivers often experience burden levels comparable to, or even exceeding, those of the patients themselves [35]. While previous review of meaning‐centered interventions have shown promise in improving depressive symptoms, the evidence remains mixed; only one of two relevant studies reported statistically significant improvements [16]. eMCP‐C's significant reductions in both depression and anxiety represent a substantive advance. This suggests that cultural adaptation and caregiver‐specific tailoring of the meaning‐centered approach may enhance efficacy in addressing core dimensions of caregiver distress within the Chinese context. Notably, we observed significant improvements in self‐esteem sustained across both assessment points. Given established evidence that diminished self‐esteem correlates with greater caregiver burden [36] and that low caregiving confidence predicts heightened psychological distress [37], these gains in self‐perception may constitute a key therapeutic mechanism through which eMCP‐C mediates its beneficial effects on depression and anxiety symptoms. Importantly, the three core therapeutic mechanisms identified in our qualitative analysis directly explain these outcomes. First, the establishment of a supportive Therapeutic Alliance provided the psychological safety necessary for emotional processing, which likely underpinned reductions in anxiety and depressive symptoms. Second, the process of Meaning‐making Through Acceptance and Action allowed caregivers to reframe existential distress into purpose‐driven engagement, directly fostering the observed improvements in spiritual well‐being (meaning/peace). Finally, the theme of Empowering Through Affirmation and Self‐Determined Action aligns with the sustained gains in self‐esteem, as strength‐based validation helped restore personal agency and caregiving competence.

Beyond alleviating psychological symptoms, eMCP‐C demonstrated preliminary positive impacts on existential well‐being. Participants exhibited substantial improvements in the meaning subscale of spiritual well‐being at both immediate post‐intervention and 3‐month follow‐up assessments—a finding aligned with previous review of meaning‐centered interventions [16]. This positive impact aligns with evidence that caregiving, though burdensome, can serve as an opportunity for meaning creation and personal growth [38]. Furthermore, while peace scores showed no immediate change, they revealed significant enhancement specifically at the 3‐month evaluation. This specific temporal pattern emerging after intervention conclusion—where meaning enhancement precedes later peace attainment—resonates with research indicating that intensive caregiving, while often painful, can simultaneously promote adaptive coping and benefit finding [19]. Within the Chinese cultural context that values harmony in adversity, this post‐intervention development of peace may reflect caregivers' gradual reconciliation of caregiving's dual nature as both burden and meaningful experience.

### 4.1 Clinical Implications

For research, this pilot study informs the design of a fully powered RCT. Concurrently, mechanistic studies should investigate the therapeutic mediators of intervention efficacy—particularly self‐esteem and meaning trajectories—through mediation analyses. Future work is warranted to examine intervention effectiveness, cost‐effectiveness, and scalability across diverse settings. Clinically, eMCP‐C demonstrates strong potential as a culturally grounded intervention for integrated psycho‐existential support in Chinese oncology settings. Its preliminary dual efficacy in reducing psychological distress (depression/anxiety) while enhancing existential well‐being (meaning/peace) and self‐esteem positions it as a comprehensive solution for existential distress among caregivers of patients with advanced cancer. The findings also carry important cultural implications for practice. The observed temporal pattern—where improvements in meaning preceded later gains in peace—resonates deeply within the Chinese cultural context that values harmony and acceptance in the face of adversity. This suggests that for Chinese caregivers, finding purpose in the caregiving role may serve as a culturally congruent pathway to eventual existential peace and reconciliation. Therefore, in clinical practice with Chinese families, interventions may be most effective if they first facilitate meaning‐making—helping caregivers reframe their role as a purposeful, familial duty—as a foundation for achieving later emotional acceptance and harmony.

If proven effective, eMCP‐C can be incorporated into existing caregiver support programs to lessen the caregiver burden. Furthermore, the positive outcomes achieved by a single facilitator (a research assistant with a psychology background) who received focused protocol training—but not extensive psychotherapy certification—suggest eMCP‐C is a structured, teachable, and disseminable intervention. This indicates a promising pathway for scaling psycho‐existential support in settings with limited specialist therapists by training other healthcare personnel (e.g., nurses, social workers) with fidelity monitoring.

### 4.2 Limitations

Limitations of this study included recruitment from a single hospital, which may limit generalizability of the findings to other populations with diverse sociodemographic characteristics. Due to the pilot nature of the study, sample size in this study was relatively small, indicating this study was underpowered to determine the causal inferences about eMCP‐C's efficacy. Additionally, there was only one eMCP‐C facilitator who was not a licensed therapist, limiting generalizability across different facilitators. However, this aspect also points to a practical strength as the facilitator's successful delivery, confirmed through fidelity checks, suggests that with standardized training and supervision, effective implementation may not be restricted to specialist therapists. This enhances the intervention's potential for broader dissemination into routine care. A future full‐scale trial could employ more than one therapist to confirm generalizability. Finally, reliance on self‐reported measures introduces the possibility of response bias, particularly given the culturally sensitive nature of psychological distress in Chinese populations. Future studies should incorporate objective biomarkers (e.g., EEG for neural correlates of distress) alongside psychometric instruments to triangulate findings.

## 5 Conclusions

eMCP‐C appeared to be feasible, acceptable, and safe among Chinese caregivers of patients with advanced cancer. Preliminary effectiveness was demonstrated in depression, anxiety, meaning and peace subscale, as well as self‐esteem after the intervention. The findings strongly support the continued evaluation of eMCP‐C through a fully powered multisite RCT to confirm the intervention effect.

## Funding

This study was funded by NICHE Research Grant 2023/2024. The funders had no role in the design and conduct of the study; collection, management, analysis, and interpretation of the data; preparation, review, or approval of the manuscript; or decision to submit the manuscript for publication.

## Conflicts of Interest

Dr. Applebaum reports relationships with PsyOnc Partners LLC, and Roon. Other authors declared no conflict of interest.

## Supporting information


Supporting Information S1

## Data Availability

The data that support the findings of this study are available from the corresponding author upon reasonable request.
