# Supplementary material for: Efficacy of an Online Meaning‐Centered Psychotherapy in Caregivers of Advanced Cancer (eMCP‐C): A Mixed‐Method Pilot Randomized Controlled Trial
Source: Psychooncology. 2026 Mar 10;35(3):e70420. doi: 10.1002/pon.70420 (PMC12974551; doi:10.1002/pon.70420)
Supplement: Supplementary file 1 — Supporting Information S1 [file PON-35-e70420-s001.docx]

**Appendix 1. Meaning-Centered Psychotherapy for Cancer Caregivers (MCP-C) protocol**

| **Session** | **Session Title** | **Overview** | **Homework reflection** | **Goals** | **Adaptations made during Cultural Adaptation Process** |
| --- | --- | --- | --- | --- | --- |
| 1 | Introduction and Sources of Meaning | 1. Introduction  2. Caregiving story  3. Definitions of meaning  4. Definitions of caregiver  5. Experiential exercise: Meaningful Moments  6. Session wrap-up | - Questions on Identity and Caregiving  - Optional reading: Copies of Man’s Search for Meaning. | Learn caregiver’s caregiving story and introduce concepts and sources of meaning | Refined handout to simplify the definitions of different sources of meaning.  Therapists will tell and capture the main points of Frankl’s story verbally. |
| 2 | Cancer Caregiving and Meaning: Identity before and after becoming cancer caregiver | 1. Process Session 1  2. Discussion of caregiving and identity  3. Experiential exercise: Identity and Caregiving  4. Session wrap-up and homework | Questions on Life as a Legacy (Exercise to be covered in Session 3) | Develop a general understanding of one’s sense of identity and the impact of being a cancer caregiver has made upon it. | Handout used for the discussion of identity before and after becoming a cancer caregiver was refined to show the relationship of cancer patient and caregiver, and also the potential changes that led to suffering.  In defining ‘identity,’ examples were added, including ‘family,’ ‘roles,’ ‘duties and responsibilities,’ ‘beliefs,’ and ‘values’  In defining ‘accomplishment,’ emphasis was added on ‘dream,’ ‘achievements,’ and ‘things you are good at’ |
| 3 | Historical Source of Meaning: Life as a Living Legacy | 1. Process Session 2  2. Discussion of life as legacy that has been given (past), one lives (present), and gives (future)  3. Experiential exercise: Life as a Legacy  4. Session wrap-up and homework | - Questions on Encountering Life’s Limitations (Exercise to be covered in Session 4)  - Share your legacy | Develop an understanding of caregiver’s legacy through exploration of three temporal legacy modes: the legacy that’s been given from the past, the legacy that one lives in the presents, and the legacy one will leave in future. | Experiential exercise was refined where the Present was integrated with the discussion of Past and Future meaning for simplification and emphasis on connectedness.  Used cultural idiom, “passing the torch” (薪火相傳) to convey Confucian value of continuity. |
| 4 | Attitudinal Sources of Meaning: Encountering Life’s Limitations | 1. Process Session 3  2. Discussion of confronting limitations and challenges associated with caregiving  3. Experiential exercise: Facing life’s limitation  4. Session wrap-up and homework | Questions on Creativity, Courage, and Responsibility (Exercise to be covered in Session 5) | Explore one of Frankl’s core therapeutic principals, that ultimately, we have the freedom and capacity to choose our attitude toward suffering and life’s limitations and to derive meaning from that choice. | The question “what would your loved one with cancer consider to be a good death?” was made optional depending on the situation of the patient’s prognosis.  Used cultural idiom “life begets life without end” (生生不息) to convey the enduring and expansive nature of legacy.  Concrete examples were given for the Legacy Project |
| 5 | Creative Source of Meaning: Engaging in Life Fully | 1. Process Session 4  2. Discussion of creativity, courage and responsibility  3. Experiential exercise: Creativity, Courage, and Responsibility  4. Session wrap-up and homework | - Questions on Connecting with Life (Exercise to be covered in Session 6)  - Self-Care Project | Develop an understanding of the significance of “creativity” and “responsibility” as important sources of meaning in life. | Emphasis was added on ‘giving to the world’ when defining the concept of creative source of meaning. |
| 6 | Experiential Sources of Meaning: Connecting with Life | 1. Process Session 5  2. Discussion of experiential sources of meaning, such as love, nature, art, and humor  3. Experiential exercise: Love, beauty, Humor  4. Session wrap-up and homework | - Questions on MCP-C Experience: Reflection and Feedback (Exercise to be covered in Session 7) | Foster an understanding of the significance of connecting with life through experiential sources of meaning-particularly through experiencing love, beauty, and humor. | Emphasis was added on ‘receiving from the world’ when defining the concept of experiential source of meaning.  Reframed the concept of humor as “self-mockery” (自嘲), “mutual teasing” (互嘲), and “finding joy in adversity” (苦中作樂). |
| 7 | Transitions: Reflections, and Hopes for the Future | 1. Transitions: Summary and reflection on previous sessions  2. Exploration of legacy and self-care projects  3. Caregiver’s experience in MCP-C: reflection and feedback  4. Closure |  | Review the sources of meaning. Review of the Legacy Project. Reflections on the lessons and impact of the therapy, discussion of hopes for the future, and the transition from being in the therapy to enacting the lessons learned in daily life as the therapy comes to an end. | N/A |

**Appendix 2. Definitions of feasibility- and acceptability-related outcomes**

| **Parameters** | **Assessment method** | **Benchmark (if any)** |
| --- | --- | --- |
| Eligibility rate | The number of participants who met the inclusion criteria divided by the number of people screened for eligibility | N/A |
| Recruitment rate | The number of participants who consented to participate in the study divided by the number of participants who met the inclusion criteria | N/A |
| Attendance rate | The number of participants who have attended all seven sessions, divided by the number of intervention group participants who completed the study | ≥80% |
| Retention rate | The number of participants who completed the study divided by the number of randomised participants. | ≥80% |
| Participant satisfaction | A questionnaire rating on four key domains, (1) usefulness of treatment, (2) opinion of the therapist, (3) perceived improvement, and (4) likelihood to recommend the treatment to others. | ≥7/10 on the 10-point scale |
| Adverse events | Records in reports | None |
